# What do European shoulder surgeons think of the frozen shoulder? Results of a questionnaire survey among the members of the European Society for Surgery of the Shoulder and the Elbow and a review of the current evidence

**DOI:** 10.1530/EOR-2024-0218

**Published:** 2025-09-04

**Authors:** Anna Várnagy, Dániel S Veres, Gábor Skaliczki

**Affiliations:** ^1^ Semmelweis University, Department of Orthopedics, Budapest, Hungary; ^2^ Semmelweis University, Department of Biophysics and Radiation Biology, Budapest, Hungary

**Keywords:** frozen shoulder, adhesive capsulitis, stiff shoulder, corticosteroid injection, capsular release, movement restriction

## Abstract

- The results of our survey conducted among the members of the European Society for Surgery of the Shoulder and the Elbow is presented in this article.
- The two most important features of frozen shoulder are movement restriction and pain.
- Frozen shoulder is considered secondary if it occurs after surgery or trauma.
- Corticosteroid injections are recommended as the first choice of pharmacological therapy.
- Patient education and physical therapy are the first choice of non-surgical therapy.
- The rate of remaining symptoms was observed in less than 20% of patients.

## Introduction

Painful motion restriction of the shoulder joint is a well-known condition in clinical practice. The disease develops gradually, initially with increasing inflammation followed by restriction of active and passive movement of the shoulder joint. Several risk factors have been described, such as diabetes mellitus (1), thyroid disorders (2), and hyperlipidaemia (3), although the exact pathophysiology is still unclear (4).

wSince Duplay’s use of the term ‘periarthritis humeroscapularis’ in 1896, the disease has been referred to by many different names (adhesive capsulitis, frozen shoulder, and stiff shoulder). However, no consensus has been reached on the term or the definition, making it difficult to evaluate and compare the reported data and therapeutic results. Similarly, there is no agreement on the condition’s classification (primary versus secondary) and staging. Rather than a widely accepted therapeutic guideline, a number of treatment modalities are used with the primary aim of reducing pain, improving range of motion, and shortening the duration of symptoms.

As the lack of uniform assessment makes it difficult to select the most effective therapy, several attempts have been made to explore trends in terminology, definition, and classification (5, 6, 7, 8, 9, 10, 11).

While previous questionnaires focused on the opinion of a specific country, our aim was to investigate the principles used in all European countries. To this end, we surveyed members of the European Society for Surgery of the Shoulder and the Elbow (SECEC/ESSSE) to find out what terminology, aetiology, classification, diagnostic, and treatment strategies they consider appropriate.

## Materials and methods

A 20-question questionnaire was sent by e-mail to all ordinary members of SECEC/ESSSE in April 2022. Completion of the questionnaire was anonymous and voluntary. The survey included general questions (workplace and experience of the respondent), items on terminology, definition, classification, and the application and success rate of various non-surgical and surgical procedures.

Throughout our study, the term ‘frozen shoulder’ was used, as proposed by The International Society of Arthroscopy, Knee Surgery and Orthopaedic Sports Medicine (ISAKOS) (6).

## Statistical analysis

All statistical analyses were made with *R* statistical software (R Core Team 2022, v4.2.1) (12) using the UpSetR package for the upset plots (Gehlenborg 2019, v1.4.0) (13).

## Results

### Distribution of respondents

**Figure 1:**
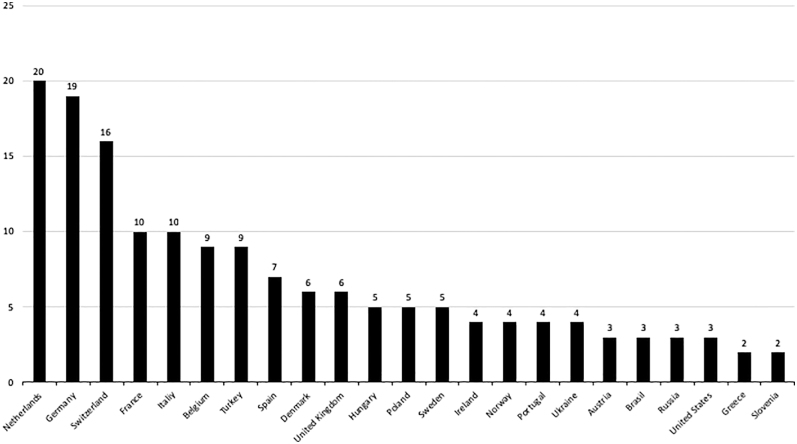
Distribution of countries of the respondents.

The survey was sent to all 863 members of SECEC, and a total of 169 completed questionnaires were collected (the response rate was 20%). The five countries with the highest number of answers were the Netherlands (20 answers), Germany (19 answers), Switzerland (16 answers), France, and Italy (ten answers each) (Fig. 1). Seventy-five percent of the respondents had been working as shoulder surgeons for at least 11 years, and 56% of them for at least 15 years. Forty percent saw more than ten patients per month with shoulder stiffness.

### Terminology, definition, and classification

**Figure 2:**
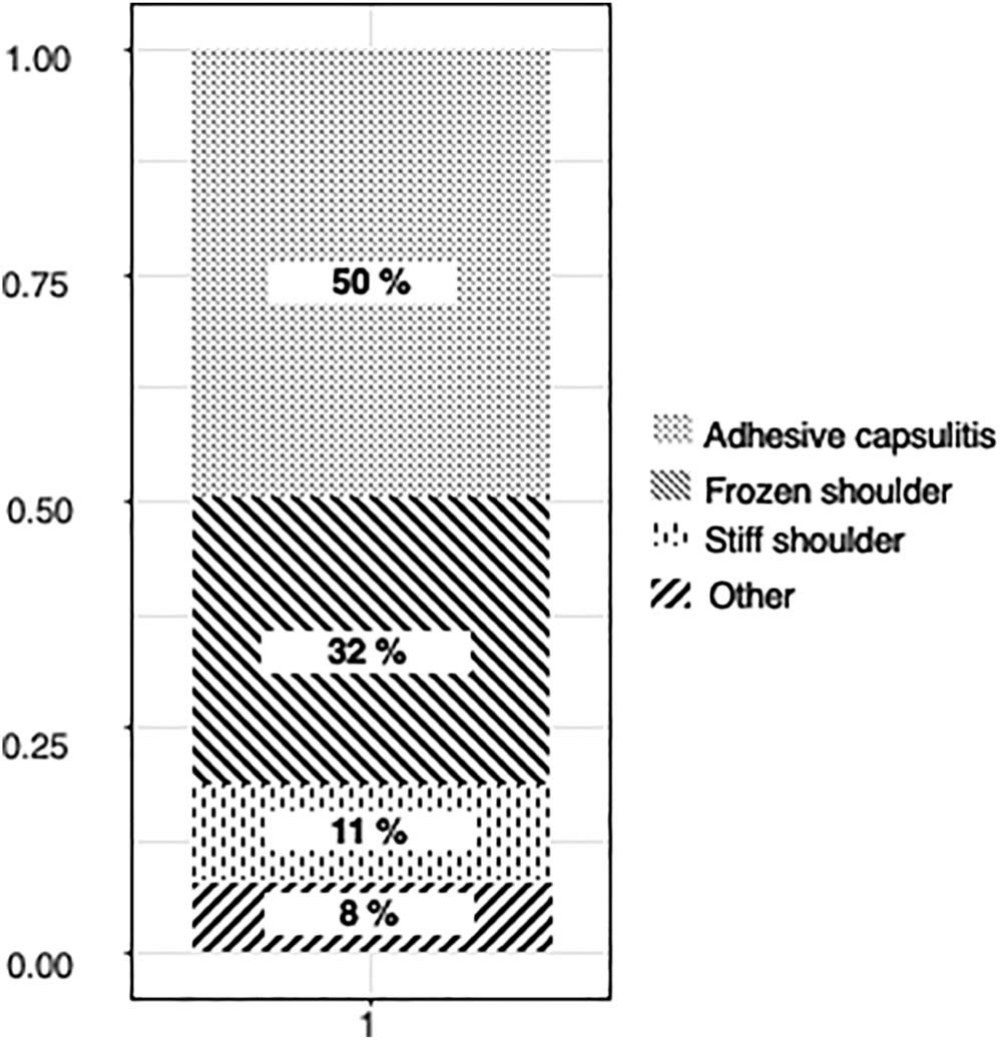
Terminology – term used by the respondents for painful movement restriction of the shoulder joint.

**Figure 3:**
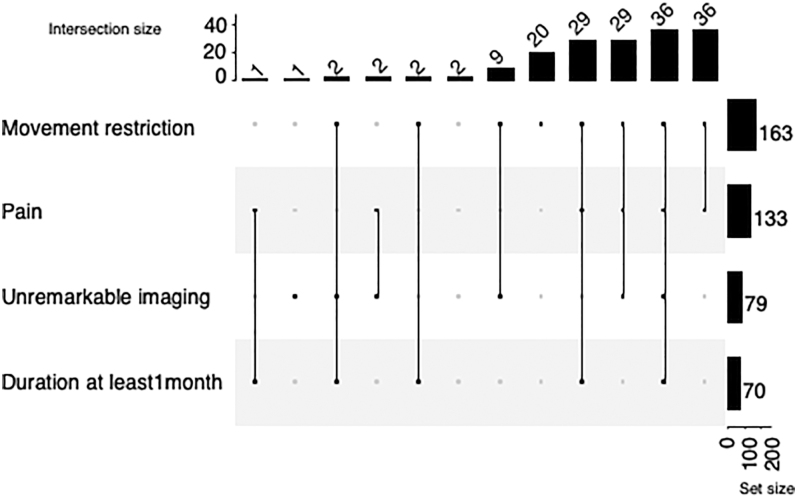
Definition – the definition had four optional elements; the rows show the frequency of each element, and the columns represent the combinations of the items.

Half of the respondents used the term adhesive capsulitis, followed by ‘frozen shoulder’, and ‘stiff shoulder’ (Fig. 2). As the most characteristic feature of the disease, the following four elements could be chosen by respondents: pain, restriction of active and passive movements, unremarkable findings on imaging, and duration of symptoms for at least 1 month. Restriction of movement (96%) and pain (79%) were the most frequently mentioned items, and the most common combination was the pair of pain and restriction of movement and all four elements (21–21%). Forty-seven percent chose unremarkable imaging findings, and 41% chose duration of symptoms for at least 1 month (Fig. 3).

Frozen shoulder is usually divided into three phases and is traditionally described as a self-limiting disease, but this latter statement has recently been questioned (14, 15). Sixty percent of our respondents agreed with the division of the disease into three phases and its self-limiting nature, while 20% accepted only the three-phase model, and 10% only the self-limiting nature. Ten percent of the respondents did not agree with any of the above statements.

**Figure 4:**
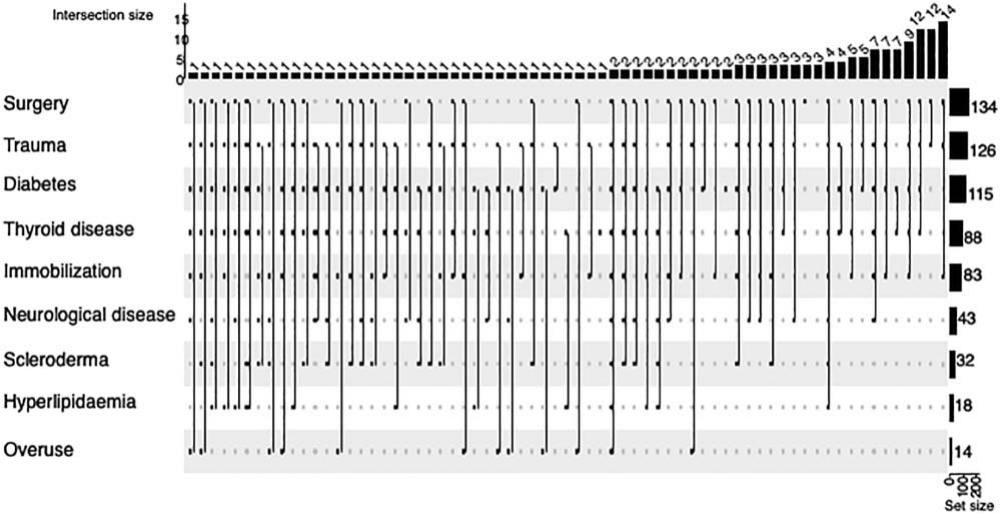
Classification – conditions in which frozen shoulder is considered to be secondary. The rows show the frequency of each possibility, and the columns show the combinations of the elements.

There is also no consensus on which cases should be considered primary or secondary frozen shoulder. In our study, most respondents regarded the condition as secondary after shoulder surgery (80%) and intra-articular trauma (75%), followed by diabetes (68%) or thyroid disease (52%), and immobilisation (49%) as a possible cause (Fig. 4).

### Diagnostics

**Figure 5:**
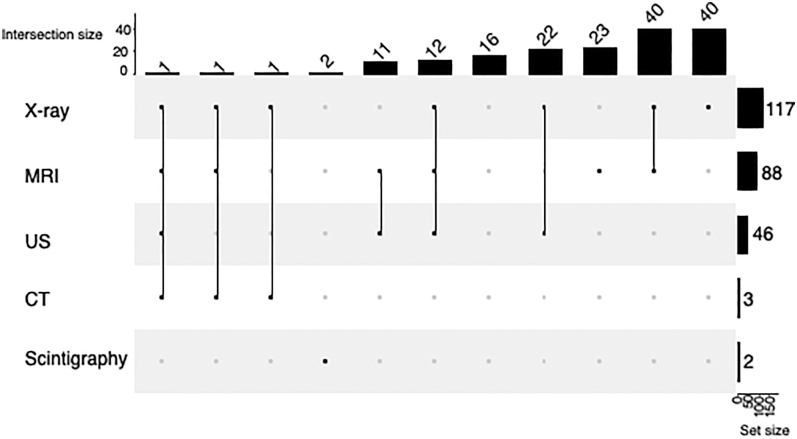
Diagnostics – the preferred imaging method for diagnosing frozen shoulder. The rows show the frequency of each element, and the columns represent the combinations of the items. MRI, magnetic resonance imaging; US, ultrasound; CT, computed tomography.

For diagnostics, we asked in a multiple-choice question about the preferred imaging methods. The most commonly (69%) used imaging was X-ray (24% by itself), followed by MRI (52%, alone or in combination with other imaging modalities) and ultrasound (27%, always in combination) (Fig. 5).

### Therapy

#### Medication and other conservative therapies

**Figure 6:**
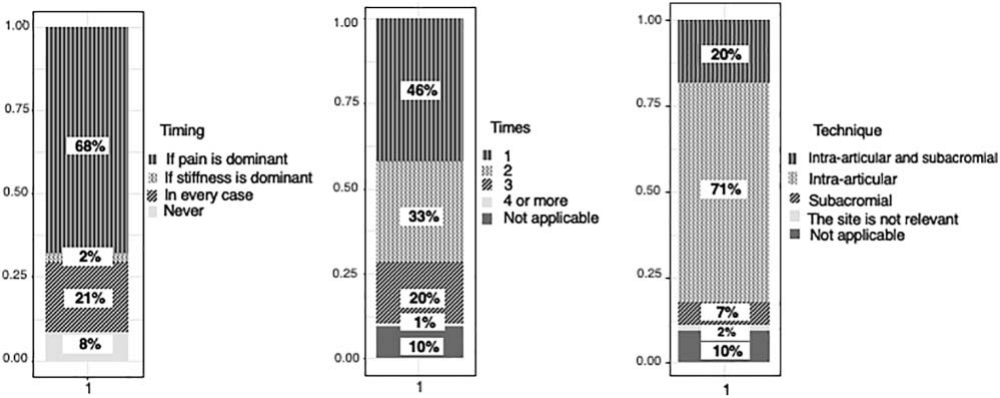
Timing, number, and technique of corticosteroid injections.

**Figure 7:**
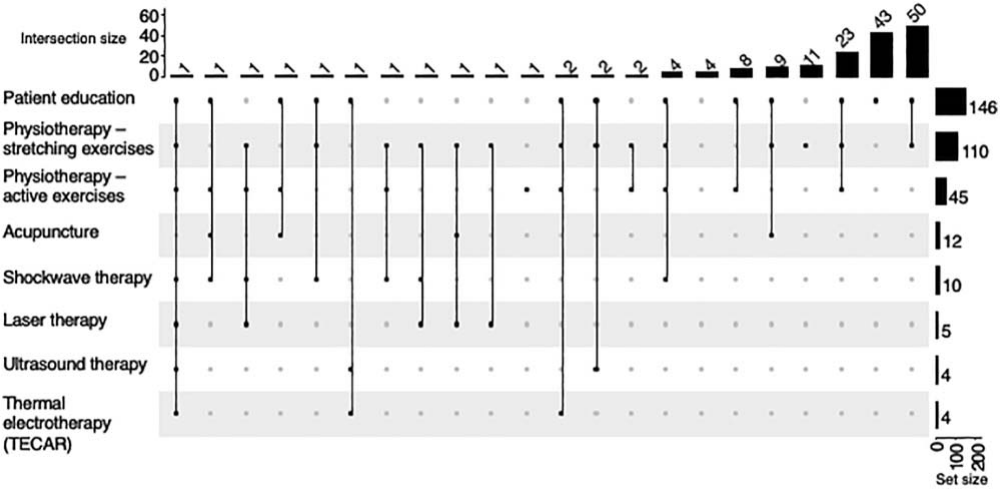
Conservative therapy – the rows show the frequency of each item, and the columns show the combinations of the elements.

Further questions focused on the different therapeutic methods. Respondents could choose from the following pharmacological options: corticosteroid injection, non-steroidal anti-inflammatory drugs (NSAIDs), oral steroids, local anaesthetics, suprascapular nerve block, hyaluronic acid injection, platelet-rich plasma, or other. Of the medications, 82% of the interviewed surgeons chose corticosteroid injections as their first choice, 20% by itself and 25% in combination with oral non-steroids. Oral non-steroids were given by 62%, and oral steroids by 29%. Corticosteroid injections were considered by two-thirds of participants in the painful phase, typically with intra-articular technique. Nearly half of the respondents applied steroid injection once, and a third of them two times (Fig. 6). Sixty percent did not require ultrasound guidance for the injection. The following non-pharmacological conservative treatment methods were available to survey respondents: patient education, physiotherapy – stretching exercises, physiotherapy – active and against resistance, shockwave therapy, acupuncture, laser, ultrasound, thermal electrotherapy (TERAC), and others. The most commonly recommended non-pharmacological conservative therapies were patient education (86%), physiotherapy involving stretching exercises (65%), and active and against-resistance exercises (26%) (Fig. 7).

#### Hydrodilatation, manipulation under anaesthesia, surgery

**Figure 8:**
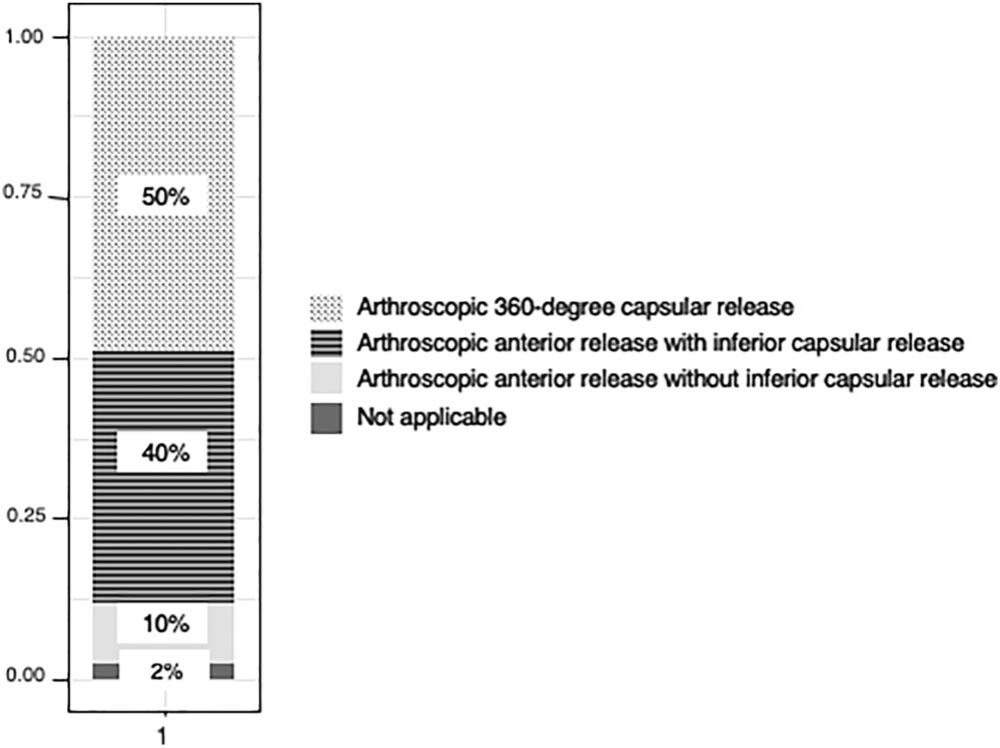
Surgical technique.

Neither hydrodilatation nor manipulation under anaesthesia is ever used by 70% of the interviewed surgeons. Attitudes on the necessity and timing of surgery were quite variable, with responses divided between the never-option and the first-choice treatment. More than two-thirds of the interviewed surgeons stated that surgery was necessary in less than 5% of all cases. For surgery, arthroscopic 360-degree capsular release (49%) and arthroscopic anterior-inferior capsular release (39%) were the preferred methods; none of the participants performed an open approach (Fig. 8). The final question related to the incidence of remaining symptoms – 78% of respondents reported less than 20% of patients with residual pain or restricted movements.

## Discussion

So far, a few attempts have been made to explore trends in the terminology, definition, and classification of frozen shoulder by conducting similar surveys, but these have either been limited to one country (e.g. Italy, Korea, Japan) (5, 7, 11) or have only covered one topic (e.g. definition) (10). To the best of our knowledge, this is the first comprehensive international survey of the definition, diagnosis, and treatment of frozen shoulder. As both the questions of previous queries and of our questionnaire differed considerably, it was unfortunately not possible to find geographical differences in general. However, for certain questions that were included both in our questionnaire and in previously published papers, we analysed how the answers depended on geographical location.

### Terminology, definition, and classification

Several authors highlighted the importance of a uniformly accepted nomenclature, but this has not been achieved so far (6, 7). The ISAKOS Upper Extremity Committee suggested that the term frozen shoulder should be used for idiopathic stiff shoulder (6); however, others use frozen shoulder and adhesive capsulitis as synonyms (16, 17). Although the name ‘frozen shoulder’ was also used throughout our questionnaire, the majority of the respondents chose the term adhesive capsulitis. This is part of a surprising trend, as it is well known from the literature that there are no adhesions present in the disease; still, the term adhesive capsulitis has become more popular over the last 40 years (18).

There are also many questions about the definition of the disease. In previous papers, there was agreement on the key characteristics of frozen shoulder (7, 10, 11), but we were curious whether our respondents agreed with the key features of the disease as defined by others. To this end, we asked specifically which of the symptoms listed in the questionnaire respondents considered most characteristic of frozen shoulder. Based on our study, movement restriction and pain are the two crucial criteria; unremarkable radiographic findings were considered relevant by less than half of our respondents. Our results are consistent with those of Bunker’s 2009 definition (18, 19), but not with the ASES (American Shoulder and Elbow Surgeon) consensus paper, where pain was not included in the diagnostic criteria (10).

Although the three-stage model and the self-limiting nature of the disease are basic tenets in the literature, they are based on typically second-source citations (18). The three-phase model was proposed by Reeves in his article in 1975 (20), but no longitudinal study has been published since then to prove it. A 2016 systematic review including only randomized controlled trials (RCTs) found that there is only low-quality evidence supporting that the disease improves without treatment (21). Nevertheless, both statements had high support in our study too. This misconception has practical implications, as it can delay the start of treatment. Mertens *et al.* proposed an alternative approach that determines the proper treatment based on the level of tissue irritability rather than the stage of the disease (22).

Primary and secondary forms of frozen shoulder are commonly distinguished, but the classification is a matter of debate. Most of our respondents categorised frozen shoulder after surgery or trauma as secondary, but it was more controversial whether the disease developing in combination with diabetes or thyroid problems is considered primary or secondary. These results are consistent with a previous study among Korean shoulder surgeons (11). It seems somewhat illogical that, although several studies have shown a link between diabetes (1), thyroid problems (2, 23), and hyperlipidaemia (3) and frozen shoulder, many people do not consider these forms to be secondary. At the same time, whether these comorbidities are termed risk factors or causes is perhaps irrelevant to treatment.

### Diagnostics

In daily practice, the diagnosis of frozen shoulder is based primarily on clinical signs and anamnesis, with imaging studies being used to rule out other diseases. The literature supports this practice: Roberts *et al.* proved that a routine X-ray is unnecessary in the diagnostic workup of frozen shoulder unless there is a relevant clinical history suggestive of serious or masquerading pathology (24). The same result is found for other imaging modalities. Although the accuracy of MRI is high in staging (25) and identifying frozen shoulder (26), neither it nor MR arthrography helps prognosis. According to Yoon *et al.*, the thickening of the capsule and rotator interval correlated with the severity of clinical symptoms and external rotation restriction, but it did not reflect the prognosis (27). In slight contrast to this knowledge, the majority of our respondents considered an X-ray to be important for diagnosis, and half of them also requested an MRI before a final diagnosis was reached.

### Therapy

#### Corticosteroid injection

Of the listed medications, most of the respondents used corticosteroid injections, typically applied intra-articularly rather than subacromially, and usually during the painful phase of the disease. This is consistent with several clinical trials that have demonstrated the effectiveness of steroid injections, both in terms of pain relief and improving range of motion (28, 29). In a recent meta-analysis of 65 studies, corticosteroid injections were found to reduce pain better than physiotherapy and placebo in the short and medium term (8 weeks and 6 months) (30). Results are inconsistent when comparing intra- and subacromial injection techniques – some papers have demonstrated the advantage of the intra-articular technique (30, 31); however, a meta-analysis found no significant difference between the two methods, noting that the subacromial technique has a lower impact on blood glucose levels, which is an important aspect for patients with diabetes (32). In terms of the site of administration, an RCT has found that the anterior approach provided better pain reduction and increased external rotation compared to a posterior approach (33). Beyond that, according to a recent meta-analysis, ultrasound-guided corticosteroid injections into the rotator interval showed significantly better results in pain relief and functional scores at 3 months compared to intra-articular injections using a posterior approach (34). Another, probably less widespread but yet effective technique could be the use of multiple site injections, as a previous study by Pushpasekaran demonstrated the advantage of the injection at three sites (subacromial, subcoracoid, posterior) over a single site, providing earlier recovery with fewer relapses (35). Multisite injection was also more effective than single-site injection in terms of pain relief and increasing range of motion in a study by Deng (36). In view of these results, the introduction of this method into daily practice could be considered.

Forty percent of the interviewed surgeons used ultrasound guidance for injection, which is higher than that of a previous Italian survey (10%) (5). This may reflect the spread of the technique or could be the result of regional variations. There is currently no recommendation on the dosage of corticosteroids for frozen shoulder. An earlier RCT found no significant difference between the effect of high (40 mg) and low (20 mg) triamcinolone acetonide injections (37), and although the possible harmful effects of corticosteroids on tendons were observed in animal studies, the results of human investigations on this topic are unclear (38, 39).

#### Oral non-steroids and steroids

Oral non-steroidal anti-inflammatory drug (NSAID) therapy is often used in the management of frozen shoulder because NSAIDs are known to provide excellent pain relief, although high-grade evidence for their efficacy is still lacking, and previous studies have also shown that their effect is merely temporary (40, 41, 42). Despite the lack of clear evidence behind its long-term positive effect, our respondents used NSAIDs in 62 percent, which practice should be considered for change.

The effects of oral steroid therapy were published in several small cohort studies of variable quality using different steroid regimens and dosages, making it really difficult to compare their results. In a randomized, placebo-controlled, double-blind trial, a 3-week course of oral prednisolone provided better short-term results, but this effect was not significant by week 6 and was lost by week 12 (43). According to a Cochrane review, oral steroids relieved pain and improved mobility in the short term; however, these benefits disappeared after the 5th month of follow-up (44). Presumably fearing the more common side effects of steroid drugs, or knowing from the literature of their mainly transient effects, our respondents used oral steroids less frequently than NSAID drugs, at 29%.

#### Patient education and physiotherapy

Of the non-pharmacological conservative treatments, the vast majority of respondents recommended patient education and physiotherapy. It is in concordance with the findings of Jones, who has shown that the lack of relevant information and definitive diagnosis causes anxiety and confusion among patients with frozen shoulder (45). Patient education also has its benefits in improving a range of various outcomes (especially biomedical outcomes, psychological health, and psychosocial functioning) among patients with chronic diseases. Patient education and communication can be effective even without personal participation – in a previous study, regular text messages sent to patients with frozen shoulder significantly increased compliance and improved the range of flexion, and external and internal rotation compared to a control group (46).

Physical therapy is undoubtedly one of the most important treatment methods in frozen shoulder, as it has been proven by moderate and strong evidence that adequate exercises and several mobilisation techniques can reduce pain and improve function. Unfortunately, the intensity, frequency, and duration of them are still unclear because these factors varied greatly across the available studies (22, 47). Therefore, physical therapists have to choose their interventions guided by the current symptoms of the patient. In the initial phase, pain relief should be the aim, followed by a decrease of restriction as the main goal in later phases.

#### Hydrodilatation

The vast majority of the interviewed surgeons did not use hydrodilatation, probably because there is inconsistent data in the literature: despite some positive results, the efficacy of the method is still unclear. There have been previous works claiming that capsular distension has only an insignificant clinical effect and is not superior to other conservative methods (48, 49). On the contrary, according to a recent meta-analysis published by Ladermann and his colleagues, among conservative therapies (hydrodilatation vs corticosteroid injection vs physiotherapy), hydrodilatation with corticosteroid provided the best short-term pain relief and resulted in the highest improvement in the range of motion across all time frames (50).

#### Manipulation under anaesthesia (MUA)

As with hydrodilatation, our respondents rejected MUA, claiming that neither method was used in 70% of cases. This is probably because, again like hydrodilatation, MUA has a controversial perception. A review from Kraal *et al.* reported a considerable increase in range of motion, Constant Score, and reduction in pain after MUA (51); however according to many other studies, only limited, low-quality, and inconsistent evidence exists on the efficacy of the method (52, 53). The main concern of MUA is the risk of iatrogenic injuries, such as labrum tears, bone bruises, rotator cuff injury, brachial plexus injury, or fracture. The results of the published imaging studies are somewhat controversial. Atoun *et al.* assessed the integrity of the rotator cuff by ultrasound after MUA and did not find any injury (54). Sasanuma *et al.* evaluated the MRI images of 30 patients treated with MUA 1 month after the procedure and found 29 capsule tears, four labral tears, and 15 bone bruises of the humeral head (55). In Kraal’s work, the overall complication rate was 0.4%, but it was mentioned that it might have been an underestimation (51). According to a large cohort prospective study, the recurrence rate is 18%, and this risk increases to 38% among patients with type-1 diabetes mellitus (56).

The assessment of the latter two treatments (hydrodilatation and MUA) is also controversial in surveys among shoulder specialists. While the acceptance of patient education and physiotherapy is typically very high (above 80%) regardless of the region, according to recent international surveys (5, 8, 11), hydrodilatation is only used by 13% of respondents in Italy (5), but by 43% in the Netherlands and Belgium (8). MUA is performed – similarly to our results – by 35% of Italian surgeons (5), but 50.7% of the respondents in Korea (11), highlighting the major regional differences.

#### Surgical therapy

Although ACR (arthroscopic capsular release) has been shown to be effective in reducing pain and increasing range of motion (42), it is typically used if non-surgical therapy fails; accordingly, two-thirds of our respondents chose surgery after 6–12 months of ineffective conservative treatment. Nevertheless, early surgical intervention (i.e. within 6 months of the onset of complaints) might shorten the duration of symptoms, which may justify this timing of the intervention in certain cases (intolerable pain and sleep disturbance) (57). The most favourable technique in our survey was 360° capsular release, which could significantly reduce pain and increase range of motion (58); however, it might not provide further benefits compared to anterior-inferior-posterior capsular release (59). In comparison with MUA, according to a recent meta-analysis, ACR did not show significant differences in terms of VAS (pain visual analogue scale) scores, range of motion after 6 months, or complication rates (60).

A multicenter, large-cohort UK study compared two interventions (ACR and manipulation under anaesthesia) with early structured physiotherapy combined with steroid injections. All three treatments were effective in pain relief and functional improvement, but none of them was clinically superior considering potential complications and cost-effectiveness (61).

Our study has some limitations. While the number of respondents is similar to previous surveys, only 20% of SECEC members responded, so it cannot be concluded that the responses reflect the views of all members. However, three-quarters of the participants have worked as shoulder specialists for more than 10 years, reflecting the fact that the responses are from experienced colleagues. It cannot be excluded that the wording of the questions influenced the answers.

## Conclusion

According to our questionnaire, there is no consensus among European shoulder specialists on most aspects of the definition, terminology, and, especially, the treatment of frozen shoulder. This is due, among other factors, to different traditions, different personal experiences and education, and, above all, the lack of relevant, consistent literature. Despite the wide range of opinions, we found a few points where at least 75% of SESEC/ESSSE members agreed. These are:

- The two most important features of frozen shoulder are movement restriction and pain.
- Frozen shoulder is considered secondary if it occurs after surgery or trauma.
- Corticosteroid injections are recommended as the first choice of pharmacological therapy.
- Patient education and physical therapy are the first choice of non-surgical therapy.
- The rate of remaining symptoms was observed in less than 20% of patients.

Our study confirms that there is a great need to develop a uniform nomenclature and come to a consensus on the basics of the diagnosis and treatment of frozen shoulder. This would be of great help to both general orthopaedic surgeons and shoulder specialists.

## ICMJE Statement of Interest

All authors certify that they have no affiliations with or involvement in any organisation or entity with any financial interest or non-financial interest in the subject matter or materials discussed in this article.

## Funding Statement

This work did not receive any specific grant from any funding agency in the public, commercial or not-for-profit sector.

## Author contribution statement

All authors contributed to the study conception and design. Data collection was done by GS and AV. Material preparation and analysis were performed by GS and AV. Statistical analysis was performed by DSV. The first draft of the manuscript was written by AV and GS. All authors commented on previous versions of the manuscript. The final manuscript was written by AV and GS. All authors read and approved the final manuscript.
